# *Perovskia atriplicifolia* Benth (Russian Sage), a Source of Diterpenes Exerting Antioxidant Activity in Caco-2 Cells

**DOI:** 10.3390/plants14172795

**Published:** 2025-09-06

**Authors:** Marzieh Rahmani Samani, Antonietta Cerulli, Gabriele Serreli, Maria Paola Melis, Monica Deiana, Milena Masullo, Sonia Piacente

**Affiliations:** 1 Dipartimento di Farmacia, Università degli Studi di Salerno, Via Giovanni Paolo II n. 132, 84084 Fisciano, SA, Italyacerulli@unisa.it (A.C.);; 2 PhD Program in Drug Discovery and Development, Dipartimento di Farmacia, Università degli Studi di Salerno, Via Giovanni Paolo II n. 132, 84084 Fisciano, SA, Italy; 3 Unità di Patologia Sperimentale, Dipartimento di Scienze Biomediche, Università degli Studi di Cagliari, Cittadella Universitaria SS 554, 09042 Monserrato, CA, Italy; gabriele.serreli@unica.it (G.S.);

**Keywords:** *Perovskia atriplicifolia* Benth, LC-(-)ESI/QExactive/MS/MS, NMR analysis, antioxidant activity, quantitative analysis

## Abstract

*Perovskia atriplicifolia* Benth., a perennial aromatic plant widespread in Iran’s Sistan and Baluchestan region, is known for its essential oil composition, rich in aromatic and non-aromatic sesquiterpenes. To the best of our knowledge, limited information exists on the composition of its non-volatile extracts. Herein, the phytochemical investigation of the EtOH extract of *P. atriplicifolia* aerial parts was performed, guided by an analytical approach based on LC-(-)ESI/QExactive/MS/MS. This led to the identification of phenolics, flavonoids, diterpenes (mainly carnosic acid derivatives), and triterpenes. Structural elucidation was performed via NMR and HRMSMS analysis. Furthermore, considering the occurrence of diterpenes closely related to carnosic acid and carnosol, known for their antioxidant properties, the antioxidant activity of the extract (0.5–5.0 μg/mL) and selected pure compounds (0.5–25 μM; compounds **5**, **7**, **9**, **10**, **12**, **16**) was evaluated in Caco-2 intestinal cells, showing significant reduction in free radical levels. The quantitative results highlighted that the above cited compounds occurred in concentrations ranging from 1.73 to 520.21 mg/100 g aerial parts, with carnosol (**12**) exhibiting the highest concentration (520.21 mg/100 g aerial parts), followed by 1α-hydroxydemethylsalvicanol (**9**) (91.73 mg/100 g aerial parts) and carnosic acid (**16**) (88.16 mg/100 g aerial parts).

## 1. Introduction

*Perovskia* is a small genus within the Lamiaceae family, comprising nine species primarily distributed in rocky regions of Central Asia, including Iran, Pakistan, and Afghanistan [1]. *P. atriplicifolia* Benth., also known as Russian sage, is one of the three native *Perovskia* spp. in Iran, particularly widespread in the Sistan and Baluchestan provinces [2,3]. Known locally in Persian as “Brazambal balochil,” this plant has been traditionally used in Iranian folk medicine to treat fevers [4], leishmaniasis [5], headache [6], rheumatic pains [7], and toothache [4].

Phytochemical studies on *Perovskia* species have identified the presence of rosmarinic acid, other hydroxycinnamic acids, monoterpenes (in essential oils), and various diterpenoids—mainly from the abietane class—including carnosol and rosmanol in the aerial parts, and tanshinones in the roots [8,9]. These compounds are associated with a range of pharmacological activities, including antioxidant, cytotoxic, neuroprotective, anti-inflammatory, and antimicrobial effects [10,11,12].

Previous investigations on *P. atriplicifolia* were mainly focused on the chemical composition of its essential oil, characterized using GC-MS [13]. To the best of our knowledge, little is known about the chemical composition of the non-volatile extracts of *P. atriplicifolia*. Herein, the phytochemical investigation of the ethanol (EtOH) extract of *P. atriplicifolia* aerial parts was performed, starting with an analytical approach based on LC-(-)ESI/QExactive/MS/MS. Based on the accurate masses, characteristic fragmentation patterns, retention times, and comparison with the literature data on *Perovskia* spp., 19 compounds (diterpenes, flavonoids, phenolics, and triterpenes) were tentatively identified. To unambiguously characterize these compounds and to distinguish among structural isomers, isolation and structure elucidation by 1D- and 2D-NMR experiments of isolated compounds were carried out. Considering the well-documented antioxidant properties of diterpenes related to carnosic acid and carnosol [14,15], the antioxidant activity of the EtOH extract and more representative diterpenes was evaluated in Caco-2 intestinal cells. With the aim of correlating the activity of the *P. atriplicifolia* aerial parts EtOH extract with the amount of the compounds present, the quantitative determination of the main constituents was carried out by an analytical approach based on LC-(-)ESI/QExactive/MS/MS.

## 2. Results

### 2.1. Chemical Investigation of the P. atriplicifolia Aerial Parts EtOH Extract

**Figure 1:**
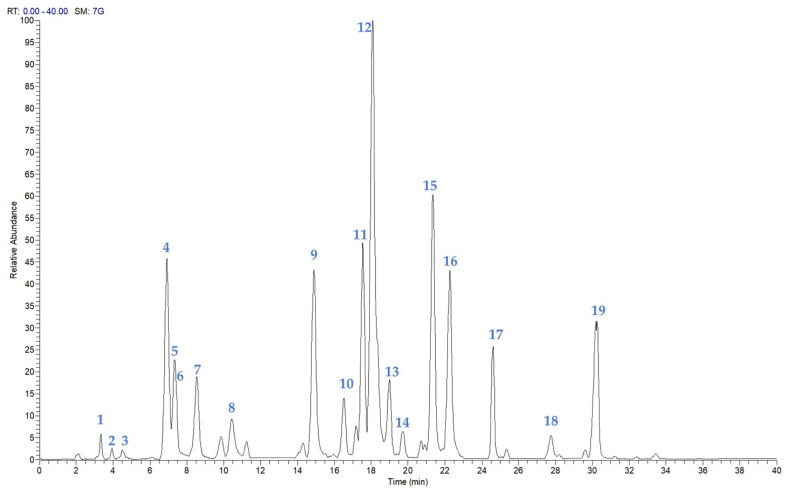
LC-ESI/HRMS analysis of EtOH extract of *P. atriplicifolia* aerial parts in negative ion mode.

An analytical approach based on LC-(-)ESI/QExactive/MS/MS, in negative-ion mode, was carried out to obtain a chemical profile of the EtOH extract of *P. atriplicifolia* aerial parts (Figure 1). The LC-ESI/HRMS analysis of the EtOH extract showed the presence of 19 compounds. Initially, some peaks were tentatively identified based on their accurate masses, on the characteristic fragmentation pattern, and by comparing the results obtained with the data reported in the literature [16] (Figure 1). The careful analysis of the multistage mass spectra suggested the presence of phenolics (**1**, **2**), diterpenes (**3**, **5**, **7**, **9**–**12**, **14**–**17**), and flavonoids (**4**, **6**, **8**), as well as triterpenes (**13**, **18**, **19**) (Table 1).

Compounds **1** and **2** showed typical molecular formulae and fragmentation patterns observed for compounds belonging to the phenolic class. Compound **1** showed an ion at *m/z* 151.0392, supporting the molecular formula C_8_H_8_O_3_, and it was tentatively identified as *p*-methoxybenzoic acid, previously reported in *P. artiplicifolia* [17]. Compound **2** was identified as caffeic acid ethyl ester, reported in *Satureja bachtiarica* [18].

The MS/MS spectra of compounds **3**, **5**, **7**, **10**–**12**, and **14**–**17** showed a main fragment ion [(M − CO_2_) − H]^−^ corresponding to a typical fragment generated by decarboxylation, in agreement with the literature data for carnosic acid derivatives [19]. Also, compound **3** showed a fragmentation pattern typical of abietane derivatives, molecules previously reported in *P. artemisioides* roots [20]. In addition, compounds **5**, **7**, **10**, and **11** were characterized by deprotonated molecular ions derived by loss of a water molecule and a subsequent loss of a methyl radical. All these data suggested that compounds **5**, **10**, and **11** were structurally related to rosmanol, a diterpenoid differing from carnosic acid in the occurrence of a lactone function between the carboxylic acid group and the hydroxy function at C-6 and in a hydroxy group at C-7; compound **7** corresponded to epi-isorosmanol, characterized by lactone function between the carboxylic acid group and the hydroxy function at C-7 and in a hydroxy group at C-6. Compound **9** was assigned to the icetexane derivative previously identified in *P. artemisioides* aerial parts [14]. Compounds **4**, **6**, and **8** were recognized as flavonoids and identified as scrophulein, eupatilin, and genkwanin, respectively, previously reported in *P. atriplicifolia* [21,22,23]. Compounds **13**, **18**, and **19** were identified as the triterpenes maslinic acid, urs-11-en-28,13β-olide, and betulinic acid, respectively, previously reported in the literature [14,24].

### 2.2. Isolation and Identification of Compounds ***1–19***

**Figure 2:**
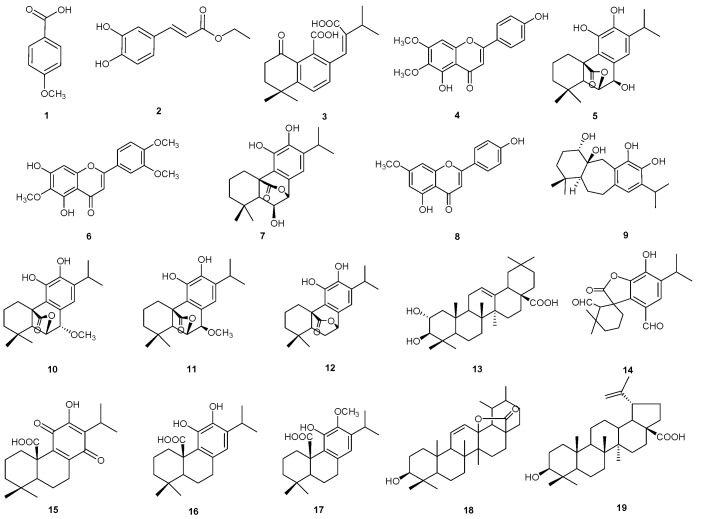
Specialized metabolites isolated from EtOH extract of *P. atriplicifolia*.

To unambiguously assign compounds corresponding to these peaks and to discriminate among structural isomers or stereoisomers, isolation and structure elucidation by 1D and 2D NMR experiments of isolated compounds were carried out. The EtOH extract of *P. atriplicifolia* aerial parts was fractionated by column chromatography on silica gel, and the fractions were purified by semipreparative HPLC-UV, as described in the Experimental Section, affording compounds reported in Table 1. The structures of the isolated compounds were established by 1D and 2D NMR spectroscopy in combination with mass spectrometry (Figure 2). The isolated compounds were identified by analysis of their NMR spectroscopic data in comparison to those reported in the literature as *p*-methoxybenzoic acid (**1**) [17], caffeic acid ethyl ester (**2**) [18], perovskin A (**3**) [20], scrophulein (**4**) [22], epirosmanol (**5**) [25], eupatilin (**6**) [23], epiisorosmanol (**7**) [14], genkwanin (**8**) [26], 1α-hydroxydemethylsalvicanol (**9**) [14], 7-*O*-methylrosmanol (**10**) [14], 7-epi-7-*O*-methylrosmanol (**11**) [26], carnasol (**12**) [14], maslinic acid (**13**) [26], rosmadial (**14**) [27], royleanonic acid (**15**) [28], carnosic acid (**16**) [14], 12-*O*-methylcarnosic acid (**17**) [14], urs-11-en-28,13β-olide (**18**) [29], and betulinic acid (**19**) [14] (Figure 2) (Figures S1–S4, S6, S7, S9, S10, S12, S13, S15–S18, S20–S25, S27 and S28, Tables S1–S3). To our knowledge, this is the first report of compounds **3**, **7**, **9**–**19** in *P. atricipifolia*. In addition, herein, compounds **15** and **18** are described for the first time in the genus *Perovskia*.

### 2.3. Evaluation of the Antioxidant Activity of EtOH Extract of P. atriplicifolia and Pure Compounds

Considering the ability of carnosol (**12**) and carnosic acid (**16**) to exert antioxidant effects by reducing ROS, NO, and lipid peroxides, as well as their involvement in activating the Nrf2/HO-1/NQO-1 pathway, where Nrf2 dissociates from Keap1, translocates to the nucleus, and binds to ARE sequences to upregulate antioxidant genes [10,30], the eventual cytotoxic and antioxidant activity of the extracts and the main abietane derivatives in the aerial part extract of *P. atriplicifolia* were evaluated.

#### 2.3.1. Cell Viability

**Figure 3:**
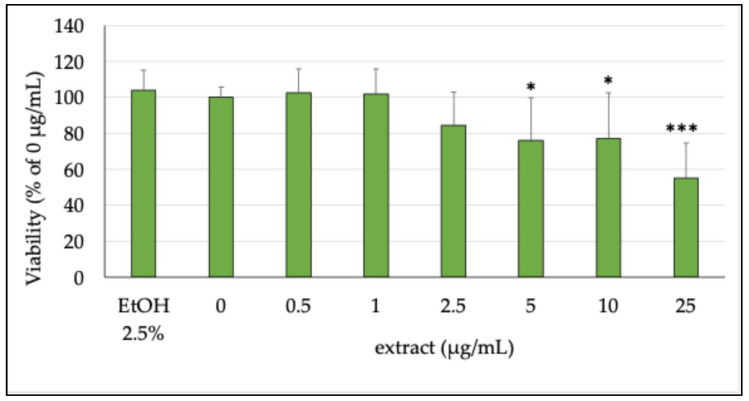
Percentage of cell viability compared with the untreated cells (0 μg/mL, 100% viable cells) of differentiated Caco-2 cells incubated for 24 h with different concentrations (0.5–25 μg/mL) of the ethanolic extract. EtOH 2.5% was tested alone as the vehicle of the extract. Each column represents the mean ± SD of the independent experiments (*n* = 12). *** = *p* < 0.001 conc. vs. 0 μg/mL; * = *p* < 0.05 conc. vs. 0 μg/mL.

The potential cytotoxic effect of the extract was assessed on differentiated Caco-2 cells, which are usually employed to simulate differentiated intestinal epithelium in vitro [31] at concentrations ranging from 0.5 to 25 µg/mL. Figure 3 reports the cell viability results obtained on differentiated cells. As shown, the tested extract caused a decrease in cellular viability of Caco-2 cells with respect to untreated cells (0 μg/mL, 100% viable cells), starting from 5 µg/mL, resulting in the lowest significantly cytotoxic concentration. This result was likely due to the very high concentration of polyphenols and certainly not from the medium used to dissolve the extract (EtOH 2.5%), as shown in Figure 3. A recent study has indeed shown that some compounds isolated from sage species, such as carnosol (**12**) and carnosic acid (**16**), can lead to toxic events at high concentrations in both cancer and normal cells [32]. Given the results obtained in this model, we then decided to use 2.5 µg/mL as the maximum concentration, because, among those tested in our experiments, it was the highest non-toxic concentration.

**Figure 4:**
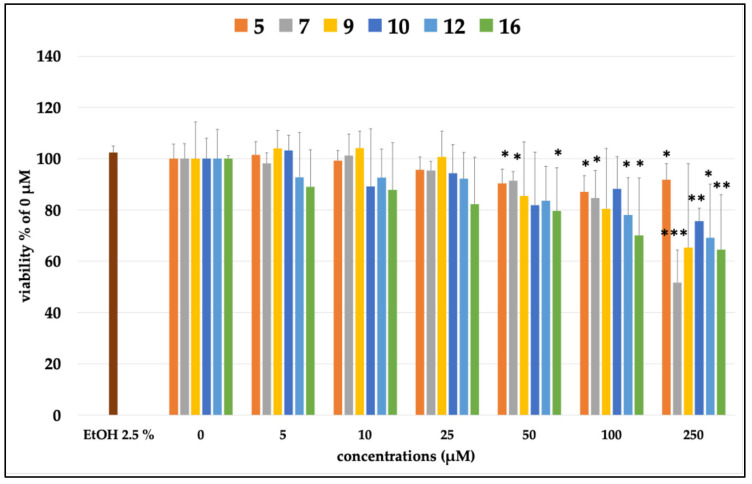
Percentage of cell viability compared with the control (0 μM, 100% viable cells) of differentiated Caco-2 cells incubated for 24 h with different concentrations (5–250 μM) of the compounds. EtOH 2.5% was tested alone as the vehicle of the compounds. Each column represents the mean ± SD of the independent experiments (*n* = 12). *** = *p* < 0.001 conc. vs. 0 μM; ** = *p* < 0.01 conc. vs. 0 μM; * = *p* < 0.05 conc. vs. 0 μM.

In this context, we also tested six of the most concentrated compounds in the extract, namely compounds **5**, **7**, **9**, **10**, **12,** and **16**. As shown in Figure 4, some of the tested compounds were found to be toxic starting at 50 µM (es, **5**, **7,** and **16**), and then increased in toxicity in a dose-dependent fashion. For this reason, in the subsequent study, only concentrations below the toxic dose were tested.

#### 2.3.2. Inhibition of ROSs Production

**Figure 5:**
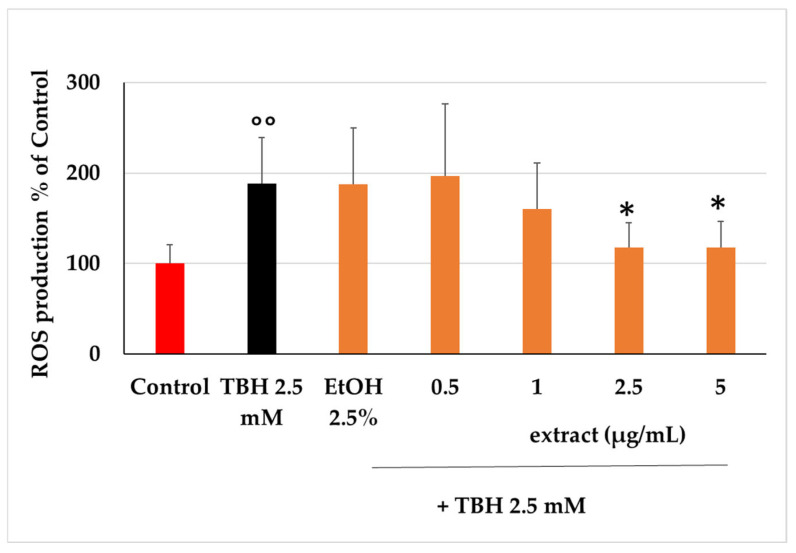
ROSs levels, measured using H_2_-DCF-DA fluorescence and expressed as a percentage of the untreated samples (Control), were assessed in Caco-2 cells after 60 min of incubation with different concentrations of the ethanol extract (0.5–5 µg/mL) in co-incubation with TBH 2.5 mM. EtOH 2.5% was tested alone as the vehicle for the compounds. Each column represents the mean ± SD of the independent experiments (*n* = 16). °° = *p* < 0.01 TBH 2.5 mM vs. Control; * = *p* < 0.05 extracts + TBH 2.5 mM vs. TBH 2.5 mM.

**Figure 6:**
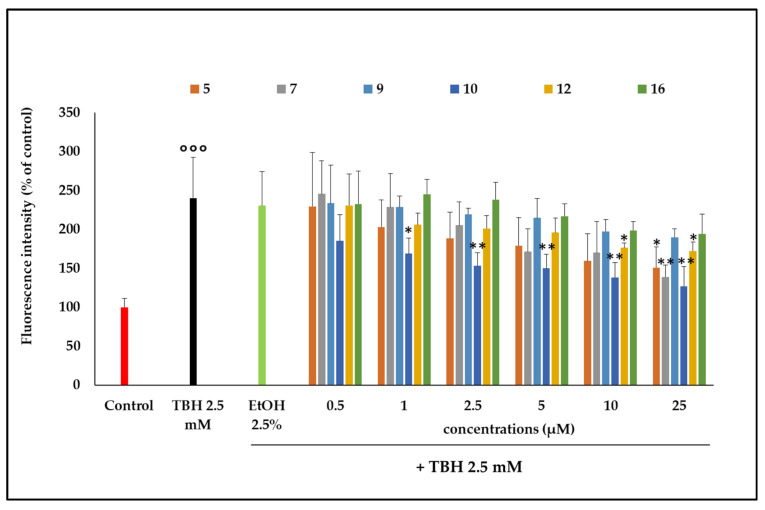
ROSs levels, measured using H_2_-DCF-DA fluorescence and expressed as a percentage of the untreated samples (Control), were assessed in Caco-2 cells after 60 min of incubation with varying concentrations of the extract (0.5–25 µM) in co-incubation with TBH 2.5 mM. EtOH 2.5% was tested alone as the vehicle for the compounds. Each column represents the mean ± SD of the independent experiments (*n* = 16). °°° = *p* < 0.001 TBH 2.5 mM vs. Control; ** = *p* < 0.01 compounds vs. TBH 2.5 mM; * = *p* < 0.05 compounds vs. TBH 2.5 mM.

TBH induced determination of intracellular ROSs production and consequent oxidative damage to cell phospholipids at the concentration of 2.5 mM, compared with untreated cells (Control, Figure 5). In cells pretreated with the ethanol extract, a significant inhibition of ROSs was detected. Although very low concentrations of extract were tested, it was able to limit the production of ROSs induced by TBH after the first 60 min of incubation. This inhibition was observed at 2.5 and 5 µg/mL after 60 min of incubation (*p* < 0.05). This outcome shows that the substances present in the extract are active in counteracting the oxidizing action of TBH, since the ethanol present in the incubation environment (EtOH 2.5%) did not show any antioxidant activity when compared with the action of TBH alone. Among the compounds involved in this activity, there could be some of the more highly represented molecules in the extract, like those already studied in the previous test. In this regard, it was seen how 7-*O*-methylrosmanol (**10**) showed antioxidant activity starting from low concentrations (1 μM) and then increasing in a dose-dependent manner (Figure 6). Other compounds, such as **12**, **5,** and **7,** showed antioxidant capacity in a dose-dependent manner starting from lower concentrations and leading to significant inhibition of TBH-induced oxidative stress at the highest concentrations tested (10 and 25 μM) (Figure 6).

### 2.4. Quantitative Analysis of the Constituents of P. atriplicifolia EtOH Extract

Quantitative analysis was conducted using an LC-(-)ESI/QExactive/MS/MS system in parallel reaction monitoring (PRM) mode, a high-resolution, high-accuracy technique for targeted quantification using quadrupole-Orbitrap hybrid instruments. In PRM, precursor ions are selected in the quadrupole, fragmented in the HCD cell, and analyzed in the Orbitrap via the C-trap, which enhances signal-to-noise by extending ion accumulation [33]. The amount (mg/100 g dry aerial parts) of the most representative abietane derivatives (**5**, **7**, **9**, **10**, **12**, and **16**) in the EtOH extract of *P. artemisioides* aerial parts (Table 2) was determined. The quantitative results highlighted that the above-cited compounds occurred in the extract in concentration ranging from 1.73 to 520.21 mg/100 g aerial parts with carnosol (**12**) exhibiting the highest concentration (520.21 mg/100 g aerial parts) followed by 1α-hydroxydemethylsalvicanol (**9**) (91.73 mg/100 g aerial parts) and carnosic acid (**16**) (88.16 mg/100 g aerial parts) (Table 2).

## 3. Discussion

### 3.1. Quantitative Analysis of Carnosol (***12***) and Carnosic Acid (***16***)

Results of quantitative analysis highlighting that carnosol (**12**) exhibited a concentration of 520.21 mg/100 g aerial parts and carnosic acid (**16**) a concentration of 88.16 mg/100 g aerial parts (Table 2) are consistent with literature reports describing carnosic acid and carnosol as key diterpenes found in the aerial parts of *Perovskia* genus, as well as in other genera within the Lamiaceae family, such as *Salvia* spp. and *Rosmarinus officinalis*. Specifically, our previous research highlighted the presence of carnosic acid and carnosol in the aerial parts of *Perovskia artemisioides*, with concentrations of 855.00 mg/100 g and 435.60 mg/100 g of dry aerial parts, respectively. Similarly, in the aerial parts of *Salvia officinalis*, these diterpenoids were also abundant, with concentrations of 829.40 mg/100 g and 115.50 mg/100 g of dry aerial parts, respectively [14]. Finally, in *R. officinalis*, during the flowering stage—which typically occurs in spring under mild weather conditions—a notable accumulation of secondary metabolites was observed, including caffeic acid, ferulic acid, and carnosic acid (729.34 mg/100 g dry aerial parts) [34]. In contrast, during the fruit maturation stage in summer, characterized by higher temperatures, the plant showed increased production of rosmarinic acid, hesperidin, and carnosol (987.01 mg/100 g dry aerial parts) [35].

### 3.2. Bioactivity of Carnosol (***12***) and Carnosic Acid (***16***)

Carnosol (**12**) and carnosic acid (**16**), the main abietane diterpenes found in the aerial parts of *Perovskia* spp., are reported to mitigate oxidative stress by reducing levels of reactive oxygen species (ROSs), nitric oxide (NO), and lipid peroxides. They also enhance antioxidant defenses by increasing glutathione (GSH) and the activity of enzymes such as superoxide dismutase (SOD), catalase (CAT), and glutathione peroxidase (GPx) [31]. These effects are associated with activation of the Nrf2/HO-1/NQO-1 pathway, wherein the compounds promote Nrf2 release from Keap1, allowing its nuclear translocation. In the nucleus, Nrf2 binds to antioxidant response element (ARE) sequences, inducing the expression of antioxidant genes [30].

Carnosic acid (**16**) and carnosol (**12**), both found also in rosemary and other sage species, have already been studied for their antioxidant effects in intestinal cells. Both compounds showed clear antioxidant activity in intestinal cells, primarily by reducing oxidative stress and enhancing cellular defense mechanisms. In particular, in pig intestinal epithelial cells, carnosol pretreatment reduced oxidative stress markers (ROSs, MDA, NO) and increased antioxidant enzyme levels (SOD). It also promoted cell survival under oxidative stress by activating the Nrf2 pathway, which upregulates antioxidant and protective genes [36]. Furthermore, studies in animal models (mice and poultry) showed that carnosic acid enhanced antioxidant capacity in the intestine. It reduced oxidative stress and inflammation, improved gut barrier function, and modulated the expression of key antioxidant proteins such as Nrf2 and SOD. These effects were observed in both chemically-induced colitis and inflammation models [37,38,39].

Oxidative stress is a major contributing factor to neurodegenerative disorders. Therefore, the neuroprotective effects observed for carnosol and carnosic acid in neuronal cells may be linked to the antioxidant properties of reported abietane derivatives [40]. Carnosic acid has been shown to exert neuroprotective activity against ischemia/hypoxia-induced injury by scavenging or reducing reactive oxygen species (ROSs) and nitric oxide (NO), and by inhibiting the COX-2 and MAPK pathways [41]. Furthermore, carnosic acid has been reported to help prevent amyloid-β (Aβ)-induced neurotoxicity, suggesting a potential role in the prevention of Alzheimer’s disease [40].

In addition, in cancer cells, carnosol inhibits proliferation, survival, migration, and invasion while promoting apoptosis. These effects are linked to the inhibition of key signaling pathways, including extracellular signal-regulated kinase (ERK), p38, c-Jun N-terminal kinase (JNK), Akt, mechanistic target of rapamycin (mTOR), and cyclooxygenase-2 (COX-2). Finally, carnosol and carnosic acid exhibit anti-inflammatory effects by activating PPAR-γ and suppressing NF-κB and COX-2 pathways [42]. They also reduce LPS-induced inflammation by inhibiting pro-inflammatory cytokines (e.g., TNF-α, IL-6) and blocking NF-κB and MAPK signaling [43].

## 4. Materials and Methods

### 4.1. Plant Material and Extraction

The aerial parts of *P. atriplicifolia* were collected from the Taftan region of Tamin, Sistan and Baluchestan Province, Iran (latitude: 28°69′ N; longitude: 61°16′ E; altitude: 2000 m above sea level), in July 2022. The plant species was identified by Dr. Mehdi Rahimmalek at the Botany Department of Isfahan University of Technology (IUT), Isfahan, Iran, using Flora Iranica [44]. The air-dried aerial parts (380 g) were ground and extracted by maceration at room temperature with EtOH (Merck, Milan, Italy) (3 × 3.6 L for 72 h).

### 4.2. LC-(-)ESI/QExactive/MS/MS Analysis

*P. atriplicifolia* EtOH extract was analyzed by hybrid quadrupole-Orbitrap mass spectrometer, using negative electrospray ionization mode (UHPLC-(-)ESI/QExactive MS/MS, Thermo Fisher Scientific, Waltham, MA, USA). An injection of 3 μL of EtOH extract (1 mg/mL) was used. LC-HRMS/MS conditions and parameters are detailed in the Supplementary Materials.

### 4.3. Isolation Procedure

The dried EtOH extract (8.15 g) was obtained by filtration and vacuum evaporation; 3 g of extract was fractionated via silica gel CC (Sephadex LH-20, GE Healthcare, Sigma-Aldrich, Milan, Italy; 100 × 5 cm) using MeOH (Merck, Milan, Italy) as the mobile phase, yielding 41 fractions (10 mL), monitored by TLC. Similar fractions (per TLC) were pooled into 8 main groups. Purification was carried out using RP-HPLC-UV, resulting in the isolation of compounds **1**–**19** (see Supplementary Materials). The purity of the tested compounds (95–99%) was determined by LC-HRMS analysis (Figures S5, S8, S11, S14, S19, and S26).

### 4.4. Cell Culture

The Caco-2 cell line was sourced from ECACC (Salisbury, UK) and handled according to the procedures described in the Supplementary Materials.

### 4.5. MTT Viability Test

To evaluate the cytotoxic effects of the extract and pure compounds on differentiated Caco-2 cells (21 days post-seeding; ECACC, Salisbury, UK), cell viability was assessed using the MTT assay, as previously described [45] (see Supplementary Materials).

### 4.6. Determination of Intracellular Reactive Oxygen Species (ROS) Production

ROSs production in Caco-2 cells was assessed using the fluorescent probe H_2_-DCF-DA, following the method of Gil et al. [46] with minor modifications (see Supplementary Materials). Extracts and pure compounds were evaluated at concentrations of 0.5–5 μg/mL and 0.5–25 μM, respectively (see Supplementary Materials).

### 4.7. Statistical Analyses

Statistical analysis was performed using GraphPad Prism 5 (GraphPad Software, San Diego, CA, USA). One-way ANOVA followed by Tukey’s post hoc test was applied, with *p*-values < 0.05 considered statistically significant.

### 4.8. Quantitative Analysis of Main Diterpenoid Derivatives (***5***, ***7***, ***9***, ***10***, ***12***, ***16***) by LC-(-)ESI/QExactive/MS/MS

Quantitative analysis was conducted using an LC-(-)ESI/QExactive/MS/MS system in Parallel Reaction Monitoring (PRM) mode, a high-resolution, high-accuracy technique for targeted quantification using quadrupole-Orbitrap hybrid instruments.

For LC-ESI/HRMS, mobile phases consisted of water (Deltek, Naples, Italy) (A) and acetonitrile (Deltek, Naples, Italy) (B), both with 0.1% formic acid, at 0.3 mL/min. Samples (5 µL, 0.25 mg/mL) were analyzed in negative-ion mode using a Luna Omega C18 column (1.6 µm, 100 × 2.1 mm; Phenomenex, Aschaffenburg, Germany) maintained at 30 °C. A linear gradient was applied, starting at 45% B and held at this % for 2.20 min; then increased to 85% B in 8.9 min, to 100% B in 0.9 min, held at 100% for 3 min, and finally returned to 45% B over 3 min.

Precursor/product ions and normalized collision energies (NCE) considered in PRM are listed in Table S1. Data were acquired in centroid mode (17,500 resolution, AGC target 1 × 10^5^, max IT 50 ms) and processed using Xcalibur 2.2 (Thermo Fisher Scientific, Waltham, MA, USA). A stock solution (1 mg/mL) of external standards was diluted in methanol to prepare six concentrations (0.5–20.0 µg/mL) for calibration. The collision cell exit potential (CXP) was 30 eV. Limits of detection (LOD) and quantification (LOQ) were determined based on signal-to-noise ratios of 3 and 10, respectively [47], as reported in Table S1 (Figure S4).

## 5. Conclusions

This work presents the first comprehensive investigation of the EtOH extract of *Perovskia atriplicifolia* aerial parts. An analytical approach based on LC-(-)ESI/QExactive/MS/MS, combined with NMR characterization of isolated compounds, enabled the identification of 19 constituents, including diterpenes, flavonoids, phenolics, and triterpenes. Among these, compounds **3**, **7**, **9**–**19** were reported for the first time in *P. atriplicifolia*. In addition, herein, compounds **15** and **18** were described for the first time in the genus *Perovskia*. The results indicate that the EtOH extract is a rich source of abietane diterpenes related to carnosic acid and carnosol. Furthermore, the extract and carnosic acid derivatives (**5**, **7**, **9**, **10**, **12**, and **16**) exhibited significant antioxidant activity, demonstrated by their ability to reduce free radical production in the Caco-2 cell line.

In particular, 7-*O*-methylrosmanol (**10**) showed antioxidant activity from as low as 1 μM in a dose-dependent manner; compounds **12**, **5**, and **7** also exhibited antioxidant activity in a dose-dependent manner beginning at lower concentrations and resulting in significant inhibition of TBH-induced oxidative stress at the highest concentrations tested (10 and 25 μM). To correlate the activity of the ethanol extract from *P. atriplicifolia* aerial parts with the levels of its constituents, a quantitative analysis of the main compounds was performed using an LC-(-)ESI/QExactive/MS/MS-based analytical approach, revealing that the identified compounds (**5**, **7**, **9**, **10**, **12**, and **16**) occurred in concentrations ranging from 1.73 to 520.21 mg per 100 g of aerial parts. Carnosol (**12**) was the most abundant (520.21 mg/100 g), followed by 1α-hydroxydemethylsalvicanol (**9**) (91.73 mg/100 g), and carnosic acid (**16**) (88.16 mg/100 g). These findings evidence the chemical richness of *P. atriplicifolia* ethanol extract, which contains some antioxidant properties, like carnosic acid and carnosol, in high amounts, highlighting its potential as an ingredient in antioxidant nutraceutical and pharmaceutical formulations.

## Figures and Tables

**Table 1:** Compounds identified in *P. atriplicifolia* aerial parts by LC-(-)ESI/QExactive/MS/MS analysis in negative ion mode.

N°	R*_t_*	[M-H]^−^	Molecular Formula	Δ ppm	MS/MS	Compound
**1**	3.42	151.0392	C_8_H_8_O_3_	1.32	136.01	*p*-methoxybenzoic acid
**2**	3.96	207.0659	C_11_H_12_O_4_	3.30	162.98, 179.03, 135.04	caffeic acid ethyl ester
**3**	4.79	329.1398	C_19_H_22_O_5_	4.50	242.15, 285.14	perovskin A
**4**	6.93	313.0718	C_17_H_14_O_6_	3.56	283.02, 298.05	scrophulein
**5**	7.33	345.1708	C_20_H_26_O_5_	3.27	283.17, 301.18	epirosmanol
**6**	7.52	343.0830	C_18_H_16_O_7_	5.19	328.05	eupatilin
**7**	8.53	345.1708	C_20_H_26_O_5_	3.36	283.17, 301.18	epiisorosmanol
**8**	10.42	283.0613	C_16_H_12_O_5_	4.38	268.04	genkwanin
**9**	14.89	333.2073	C_20_H_30_O_4_	3.91	179.11, 191.11, 315.20	1α-hydroxydemethylsalvicanol
**10**	16.47	359.1868	C_21_H_28_O_5_	4.09	283.17, 329.18, 315.16	7-*O*-methylrosmanol
**11**	17.54	359.1867	C_21_H_28_O_5_	3.84	283.17, 329.18, 315.16	7-epi-7-*O*-methylrosmanol
**12**	18.07	329.1760	C_20_H_26_O_4_	3.93	285.19	carnasol
**13**	18.99	471.3486	C_30_H_48_O_4_	3.64	453.34	maslinic acid
**14**	19.70	343.1555	C_20_H_24_O_5_	4.43	219.06, 299.16, 315.16, 325.14	rosmadial
**15**	21.33	345.1710	C_20_H_26_O_5_	3.88	286.19, 301.22	royleanonic acid
**16**	22.28	331.1916	C_20_H_28_O_4_	3.82	287.20	carnosic acid
**17**	24.61	345.2072	C_21_H_30_O_4_	3.31	283.17, 286.19, 301.18	12-*O*-methylcarnosic acid
**18**	27.73	453.3377	C_30_H_46_O_3_	3.13	407.33	urs-11-en-28,13β-olide
**19**	30.21	455.3534	C_30_H_48_O_3_	3.05	189.14, 206.99	betulinic acid

**Table 2:** Quantitative results of compounds **5**, **7**, **9**, **10**, **12**, and **16** (mg/g extract ± SD) in *P. atriplicifolia* EtOH Extract.

Compound	mg/100 g Dry Plant ± SD
**5**	20.73 ± 0.14
**7**	11.74 ± 0.39
**9**	91.73 ± 1.87
**10**	1.73 ± 0.40
**12**	520.21 ± 23.29
**16**	88.16 ± 7.26

## Data Availability

The original contributions presented in this study are included in the article/Supplementary Materials. Further inquiries can be directed to the corresponding author.

## Supplementary Materials

The following supporting information can be downloaded at: https://www.mdpi.com/article/10.3390/plants14172795/s1, Figures S1, S3, S6, S9 S12 S15, S17, S20, S22, S24, S27: ^1^H NMR Spectra (600 MHz, CD_3_OD) of compounds **3**, **5**, **7**, **9**, **10**, **11**, **12**, **14**, **15**, **16**, **17**, respectively; Figures S2, S4, S7, S10, S13, S16, S18, S21, S23, S25, S28, HRMS Spectra of compounds **3**, **5**, **7**, **9**, **10**, **11**, **12**, **14**, **15**, **16**, **17**, respectively; Figures S5, S8, S11, S14, S19, S26: LC-MS Spectra of compounds **5**, **7**, **9**, **10**, **12**, **16**, respectively; Table S1: NMR Spectroscopic Data (600 MHz, CD_3_OD) of Compounds **3**, **5**, **7**, **10**, **11**; Table S2: NMR Spectroscopic Data (600 MHz, CD_3_OD) of Compounds **12**, **15–17**; Table S3: NMR Spectroscopic Data (600 MHz, CD_3_OD) of Compounds **9**, **14**; Table S4: LC–HRMS/MS conditions for quantitation of compounds **5**, **7**, **9**, **10**, **12**, **16**.
