# Supplementary material for: Perovskia atriplicifolia Benth (Russian Sage), a Source of Diterpenes Exerting Antioxidant Activity in Caco-2 Cells
Source: Plants (Basel). 2025 Sep 6;14(17):2795. doi: 10.3390/plants14172795 (PMC12431491; doi:10.3390/plants14172795)
Supplement: Supplementary file 1 [file plants-14-02795-s001.zip › plants-3829650-supplementary.pdf]

Supplementary materials

Perovskia atriplicifolia Benth, a source of diterpenes exerting antioxidant activity in Caco-2 cells

Marzieh Rahmani Samani [1,2], Antonietta Cerulli [1], Gabriele Serreli [3], Maria Paola Melis [3], Monica Deiana [3], Milena Masullo [1], and Sonia Piacente [1,*]

[1] Dipartimento di Farmacia, Università degli Studi di Salerno, Via Giovanni Paolo II n. 132, 84084 Fisciano, SA, Italy;
[2] PhD Program in Drug Discovery and Development, Università degli Studi di Salerno, Via Giovanni Paolo II n. 132, 84084 Fisciano, SA, Italy
[3] Unità di Patologia Sperimentale, Dipartimento di Scienze Biomediche, Università degli Studi di Cagliari, Cittadella Universitaria SS 554, 09042 Monserrato, Italia;
* Correspondence: piacente@unisa.it; Tel: +39-089969763

General Methods

NMR experiments were acquired on a Bruker Ascend-600 spectrometer(Bruker BioSpin GmBH, Rheinstetten, Germany). For the isolated compounds, DQF-COSY, HSQC, HMBC and ROESY spectra were acquired in methanol-d_4 (99.95%, Sigma-Aldrich, Milan, Italy). Topspin 3.2 software was used to process the data. Semi-preparative HPLC-UV was performed with a Phenomenex Luna C18 (250 mm × 10 mm; 10μm) column on an Agilent 1260 Infinity system (Agilent Technologies, Palo Alto, CA, USA), equipped with a binary pump (G-1312C), and a UV detector (G-1314 B). Semipreparative TLC (Thin Layer Chromatography) was performed on silica gel F_{254} (Macherey-Nagel Deltek, Naples, Italy) plates (20 cm × 10 cm), mixtures of $CHCl_3$–CH_3OH–H_2O (80:18:2), and BuOH, acetic acid, and H_2O (60:15:25) (VWR international PBI S.r.l., Milan, Italy) were used as mobile phases to obtain a separate ion distance of 80 mm. Detection was carried out by spraying cerium (IV) sulphate followed by heating at 100 °C for five minutes.
LC-HRMS/MS data were acquired on ultra-high-performance liquid chromatography coupled to hybrid quadrupole-Orbitrap mass spectrometer, using negative electrospray ionization mode (UHPLC-(-)ESI/Q Exactive MS/MS).

Reagents and Solvents

Ethanol and water for extraction and HPLC grade solvents were purchased from VWR International PBI (Milan, Italy). LC-MS grade solvents and methanol-d_4 were purchased from by Merck (Milan, Italy)

LC-(-)ESI/QExactive/MS/MS Analysis

P. atriplicifolia EtOH extract was analysed by hybrid quadrupole-Orbitrap mass spectrometer, using negative electrospray ionization mode (UHPLC-(-)ESI/Q Exactive MS/MS), operating in negative ion mode. LC-HRMS was performed on a Luna C18 column (5 µm, 150 × 2.1 mm; Phenomenex, Germany) at 0.2 mL/min. A binary solvent system was used: eluent A (H_2O + 0.1% FA) and eluent B (ACN + 0.1% FA). The gradient program was: 0–5 min, 45% B; 5–28 min, 45–85% B; 38–43 min, 100% B; 43–50 min, held before re-equilibration.

A 5 µL injection of EtOH extract (1 mg/mL) was used. ESI source settings: capillary voltage −48 V; tube lens −176.47 V; source temp 280 °C; sheath/aux gas (N_2), 15/5; sweep gas 0. MS scan range: m/z 120–1400. For MS/MS, a data-dependent acquisition (DDA) with 30% collision energy was applied to top-intensity precursors. Xcalibur™ v2.2 was used for control, acquisition, and analysis.

Isolation procedure

For isolation of pure compounds, an RP-HPLC-UV system was used. Fractions were dissolved in MeOH in a concentration of 100 mg/ml. The elution gradient was obtained using water with 0.1% formic acid and acetonitrile with 0.1% formic acid as mobile phases at a flow rate of 2.0 mL/min. A Synergi Hydro RP 80A column (25 cm × 10mm, 10 µm; Phenomenex, Milano, Italy) was used. The detection wavelength was 254 nm, and the analysis was performed at room temperature.

Fractions 6, 7 and 8 were purified using the following linear gradient: 0-30 min, from 5% to 95% of B, 30-35 min at 95% of B, 35-40 min from 95% to 100% of B, 40-45 min at 100% B (injection: 20 µL for each run). In detail, from fraction 6 (20-23) (243 mg) compounds **1** (0.9 mg, t_R = 21.61 min), **12** (1.1 mg, t_R = 31.25 min), **15** (3.2 mg, t_R = 33.19 min), **16** (1.2 mg, t_R = 34.79 min), and **17** (2 mg, t_R = 35.48 min) were purified. Fraction 7 (24-27) (23.9 mg) was purified to yield compounds **2** (1.2 mg, t_R = 22.70 min), **3** (1.4 mg, t_R = 24.14 min), and **9** (3.2 mg, t_R = 33.19 min). From fraction 8 (28-41) (443.6 mg), compounds **6** (1.1 mg, t_R =18.43 min), **4** (1.4 mg, t_R =21.72 min), and **8** (1.0 mg, t_R =22.15 min) were purified.

Fraction 5 (18-19) (79.6 mg) was purified using the following linear gradient: 0-15 min, from 5% to 80% of B; 15-20 min from 80% to 93% of B; 20-23 min from 93% to 100% of B; 23-33 min was held at 100% B (injection: 20 µL for each run). In these conditions, compounds **5** (1.8 mg, t_R = 19.32 min), **7** (1.4 mg, t_R = 19.74 min), **10** (3.2 mg, t_R = 22.98 min), **11** (2.6 mg, t_R = 23.40 min), **13** (1.4 mg, t_R = 24.02 min) were isolated. Fraction 4 (13-17) (370.9 mg) was purified using the following linear gradient: from 0-15 min, from 5% to 86% of B; 15-20 min, from 86% to 93% of B; 20-33 min from 93% to 100% of B; for 10 min at 100% of B. In this way, compounds **14** (2.0 mg, t_R = 27.09 min), **18** (1.8 mg, t_R = 28.43 min), and **19** (1.2 mg, t_R = 22.14 min) were isolated. Fraction 3 (9-12) (186.1 mg) was purified using the following linear gradient: 0-5 min, from 10% to 20% of B; 5-37 min from 20% to 95% of B; 37-50 min from 95% to 100% of B; held for 10 min at 100% of B (injection: 20 µL for each run); in this way compound **19** (1.2 mg, t_R =37.98 min) was obtained.

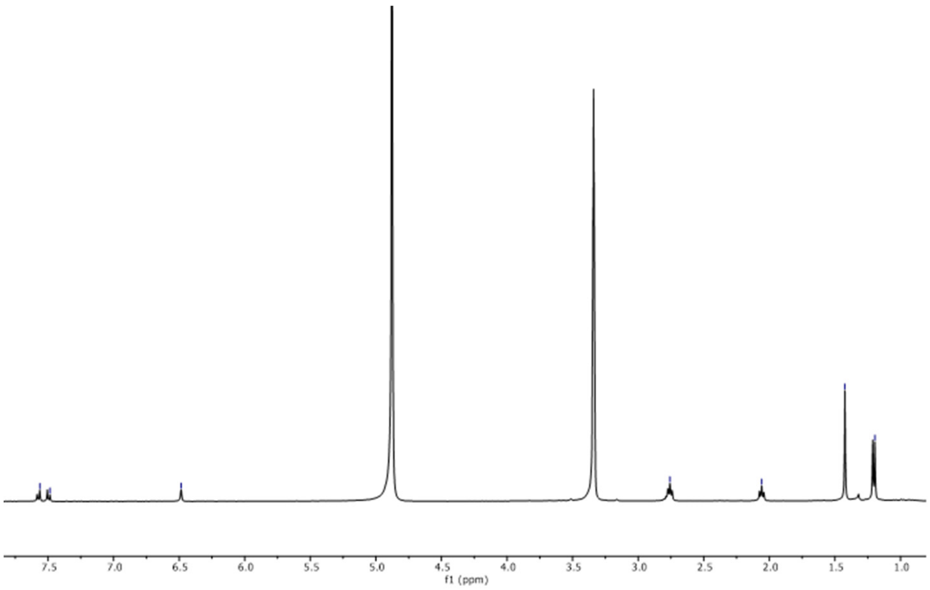

Figure S1. ^{1}H NMR Spectrum (600 MHz, CD_3OD) of compound **3**.

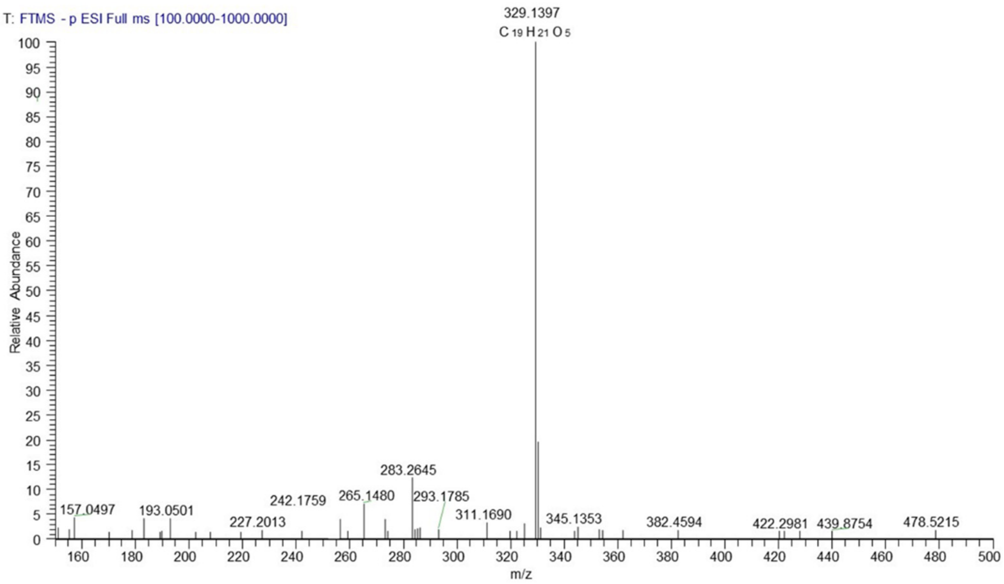

Figure S2. HRMS Spectrum of compound **3**.

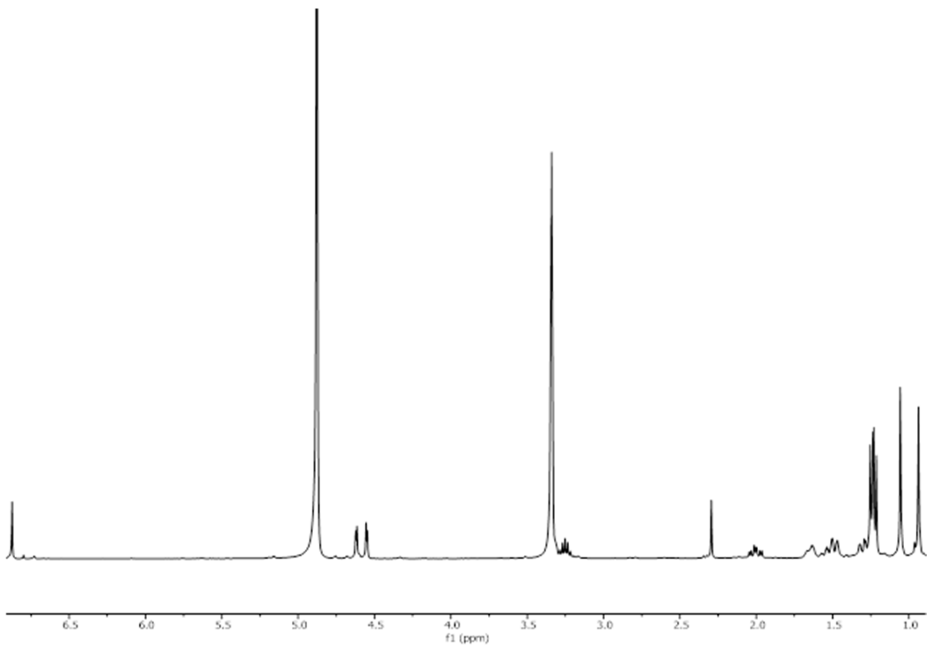

Figure S3. ^{1}H NMR Spectrum (600 MHz, CD_3OD) of compound **5**.

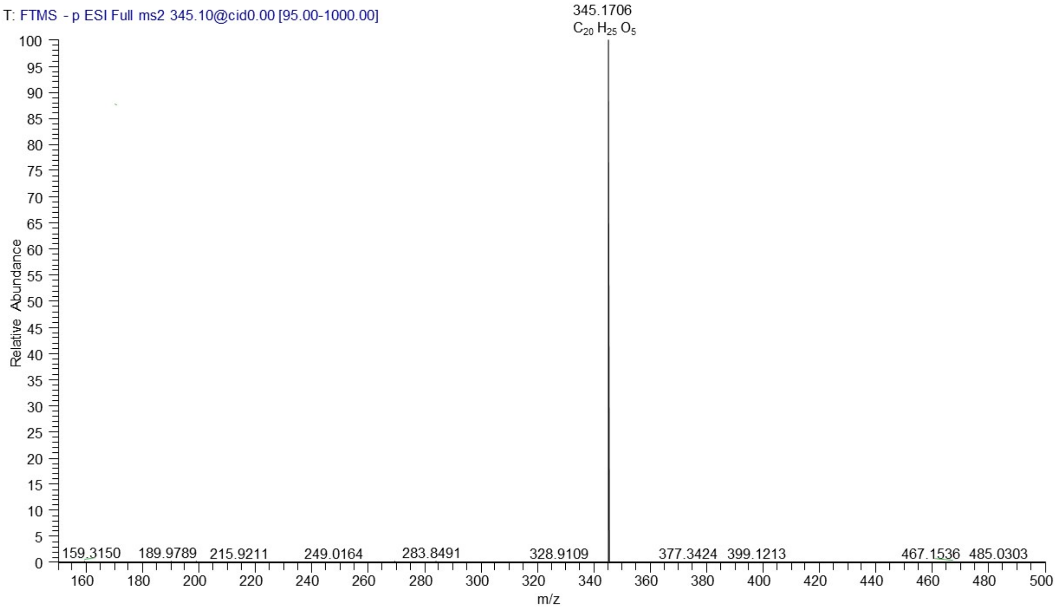

Figure S4. HRMS Spectrum of compound **5**.

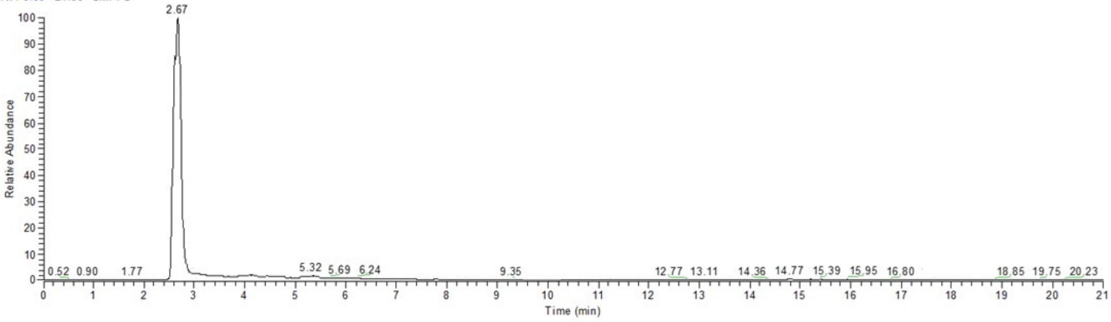

Figure S5. LC-MS Spectrum of compound **5**.

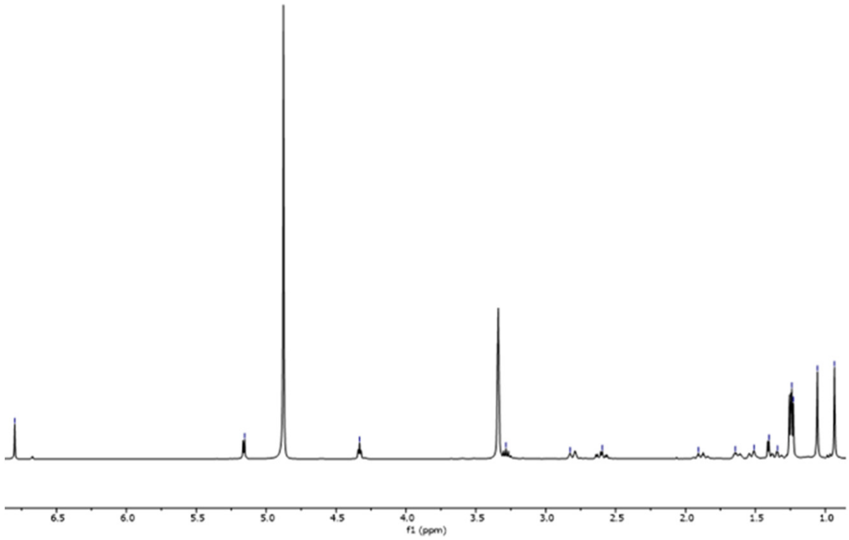

Figure S6. ^{1}H NMR Spectrum (600 MHz, CD_3OD) of compound **7**.

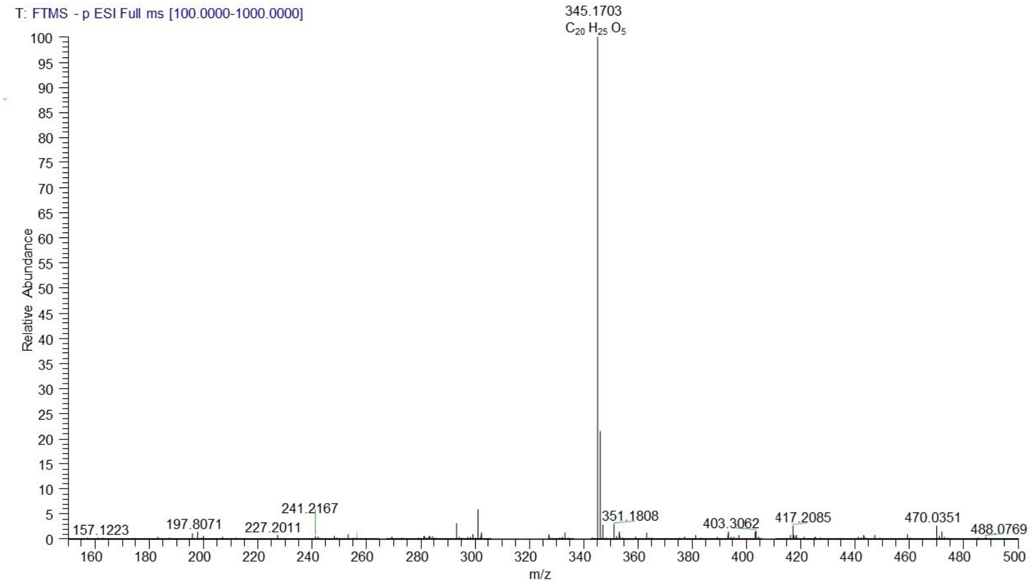

Figure S7. HRMS Spectrum of compound **7**.

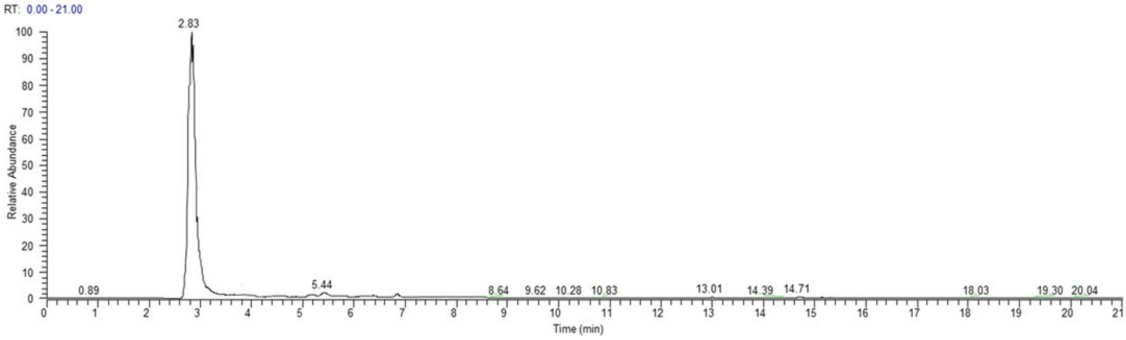

Figure S8. LC-MS Spectrum of compound **7**.

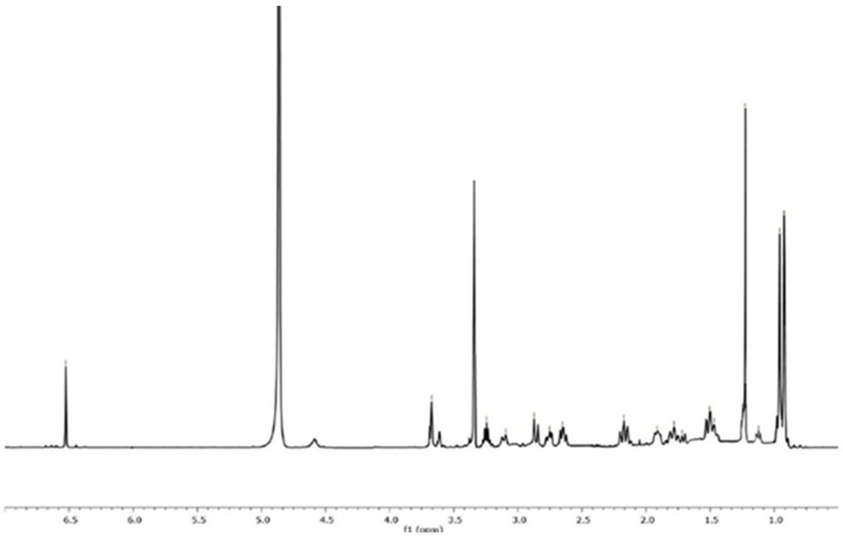

Figure S9. ^{1}H NMR Spectrum (600 MHz, CD_3OD) of compound **9**.

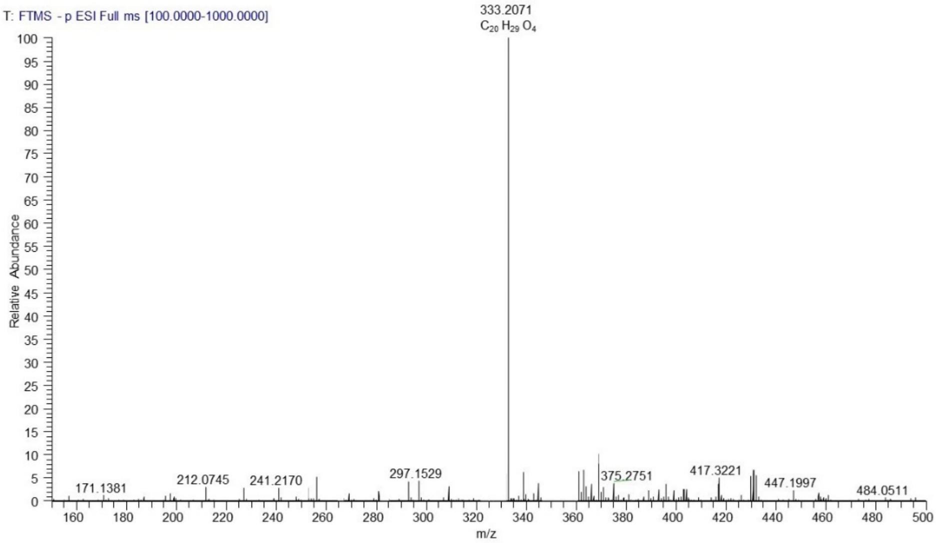

Figure S10. HRMS Spectrum of compound **9**.

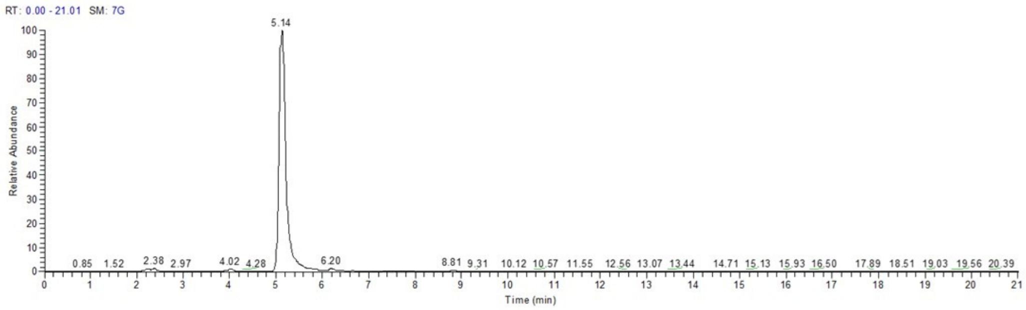

Figure S11. LC-MS Spectrum of compound **9**.

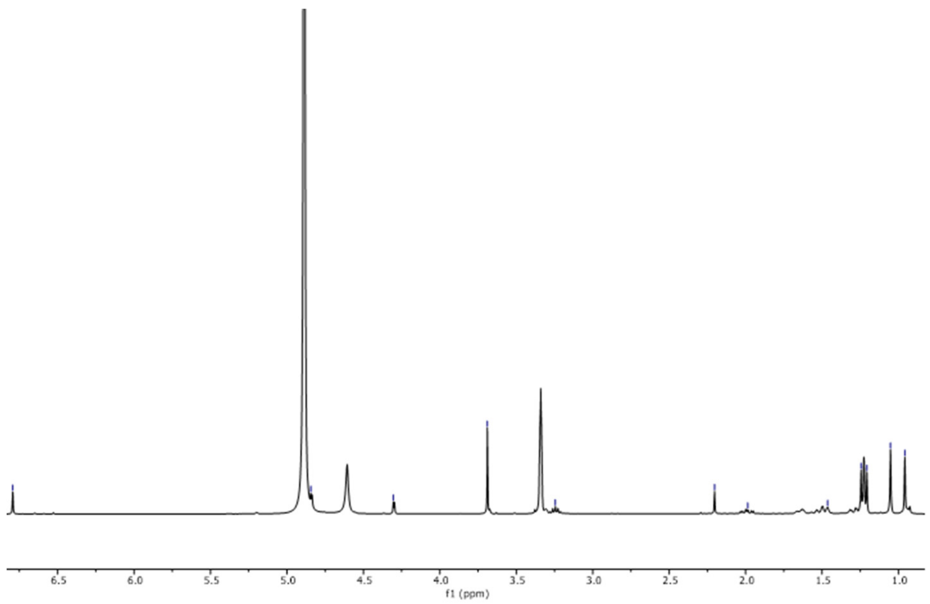

Figure S12. ^{1}H NMR Spectrum (600 MHz, CD_3OD) of compound **10**.

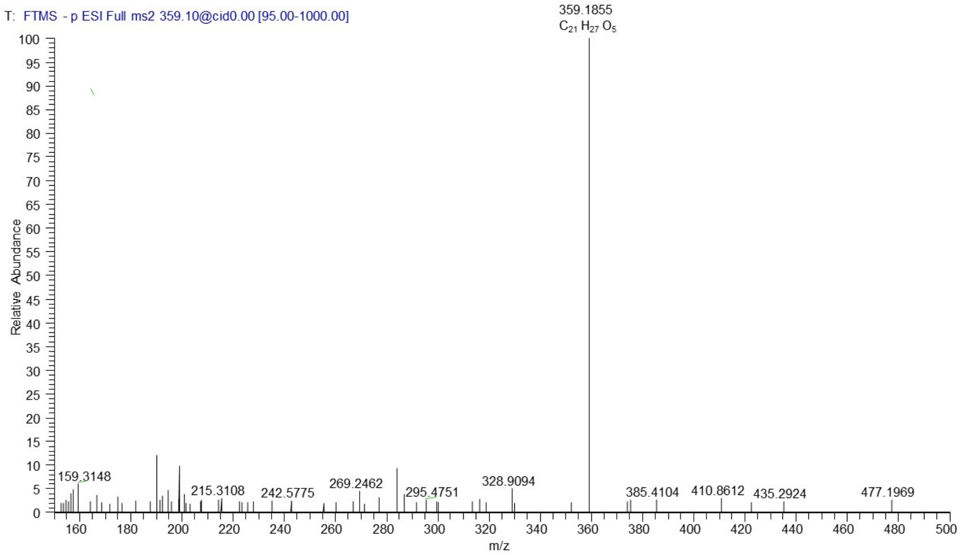

Figure S13. HRMS Spectrum of compound **10**.

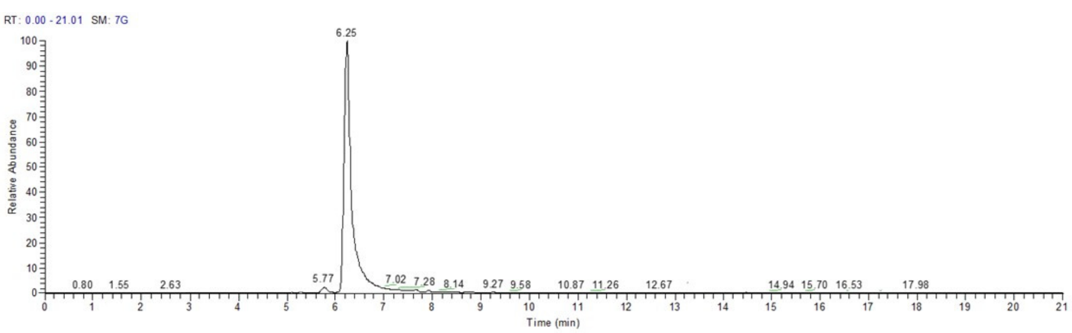

Figure S14. LC-MS Spectrum of compound **10**.

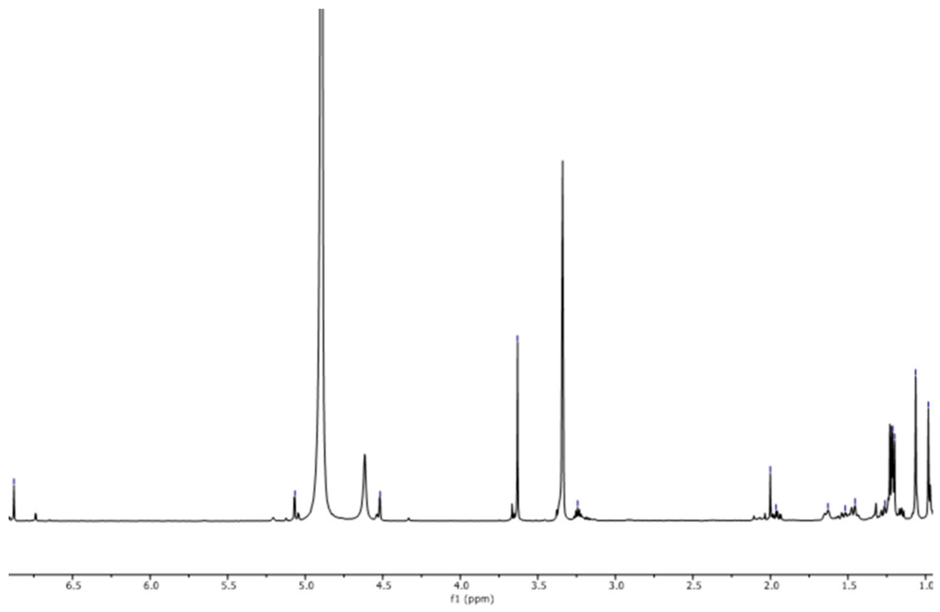

Figure S15. ^{1}H NMR Spectrum (600 MHz, CD_3OD) of compound **11**.

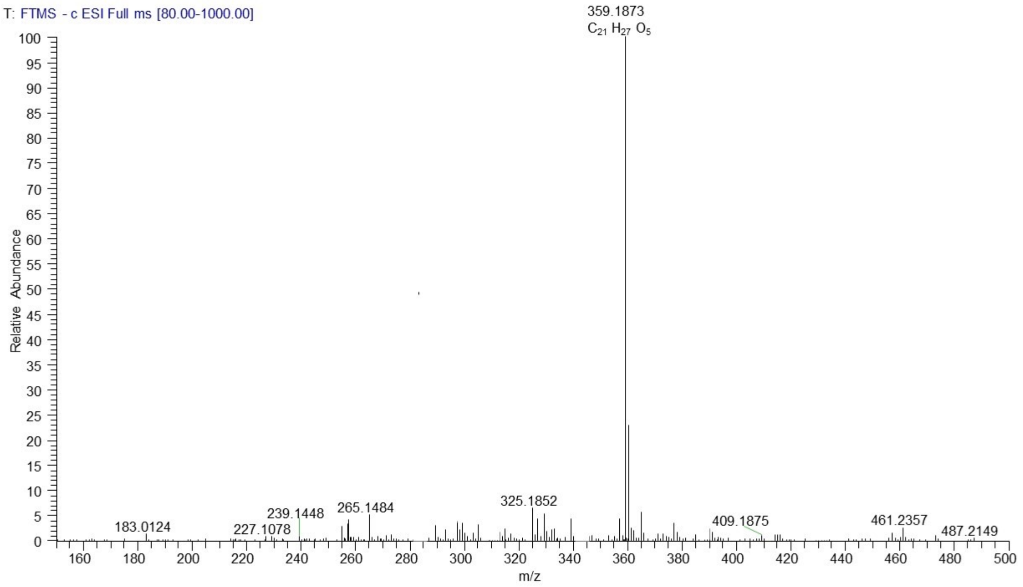

Figure S16. HRMS Spectrum of compound **11**.

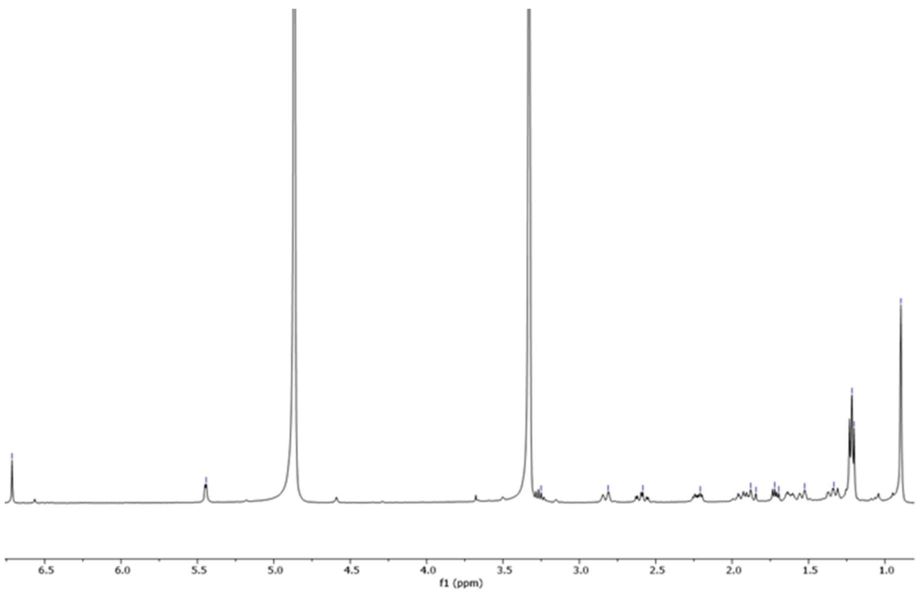

Figure S17. ^{1}H NMR Spectrum (600 MHz, CD_3OD) of compound **12.**

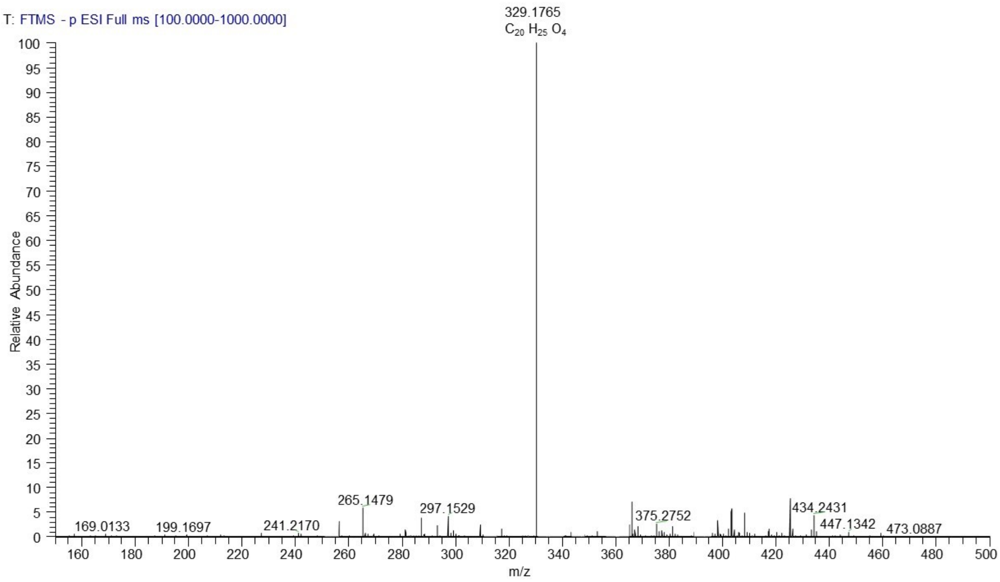

Figure S18. HRMS Spectrum of compound **12**.

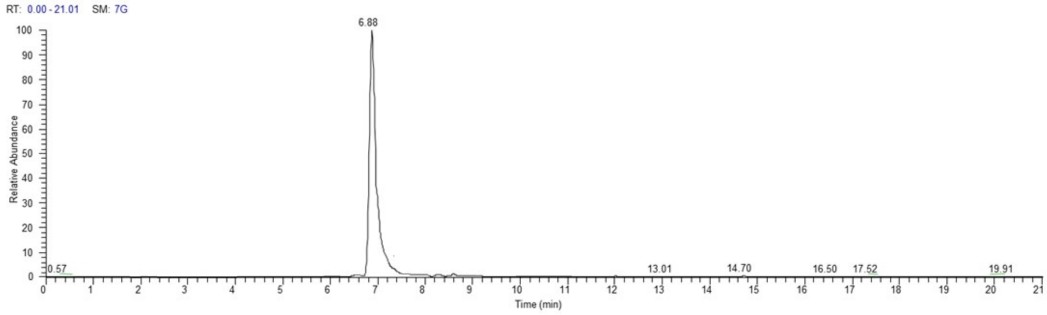

Figure S19. LC-MS Spectrum of compound **12**.

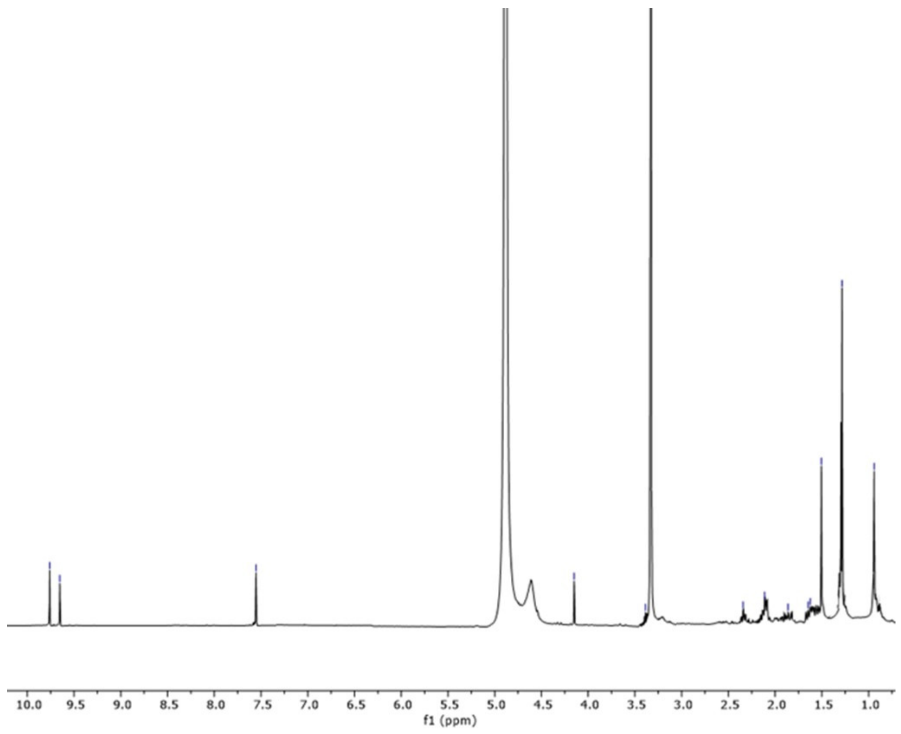

Figure S20. ^{1}H NMR Spectrum (600 MHz, CD_3OD) of compound **14.**

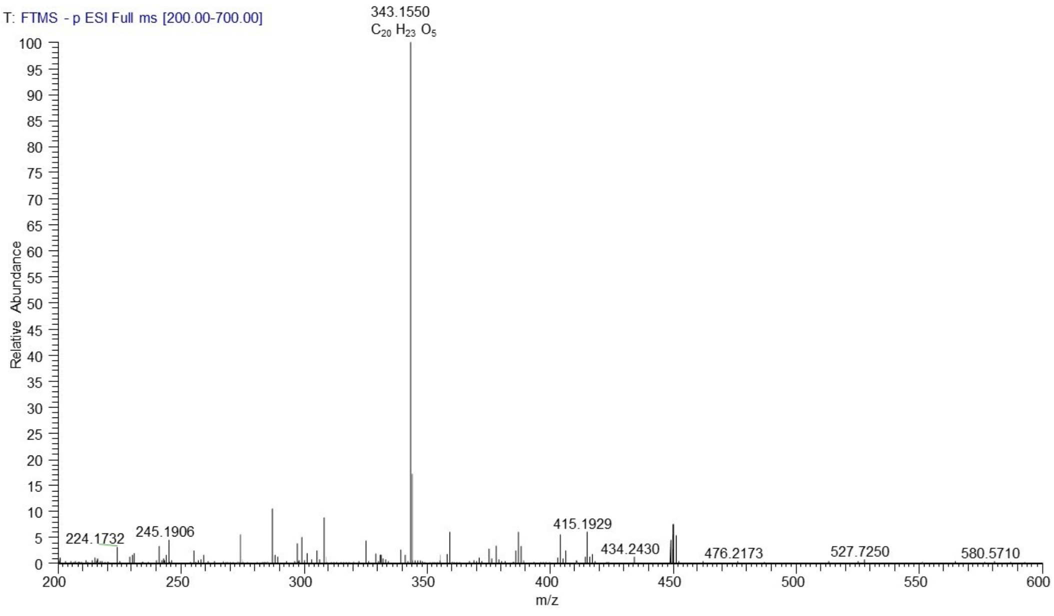

Figure S21. HRMS Spectrum of compound **14**

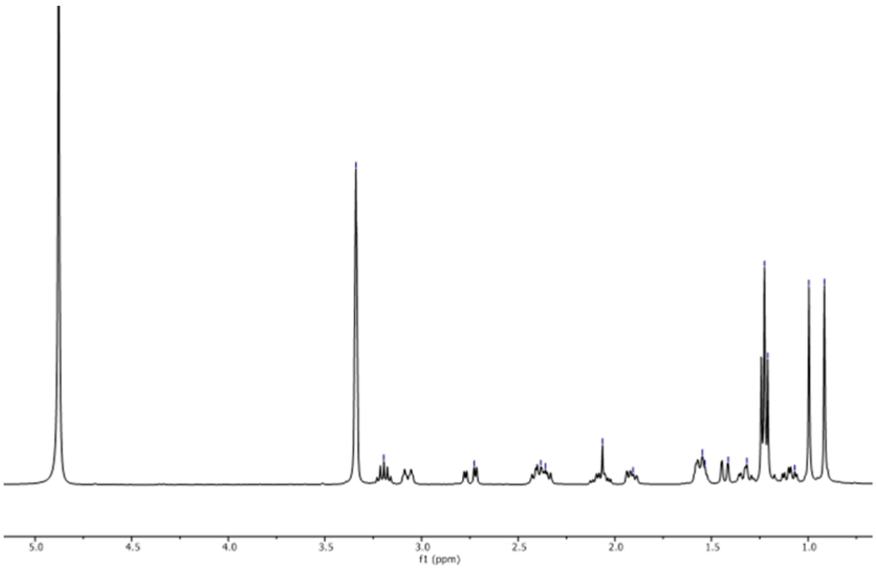

Figure S22. ^{1}H NMR Spectrum (600 MHz, CD_3OD) of compound **15**.

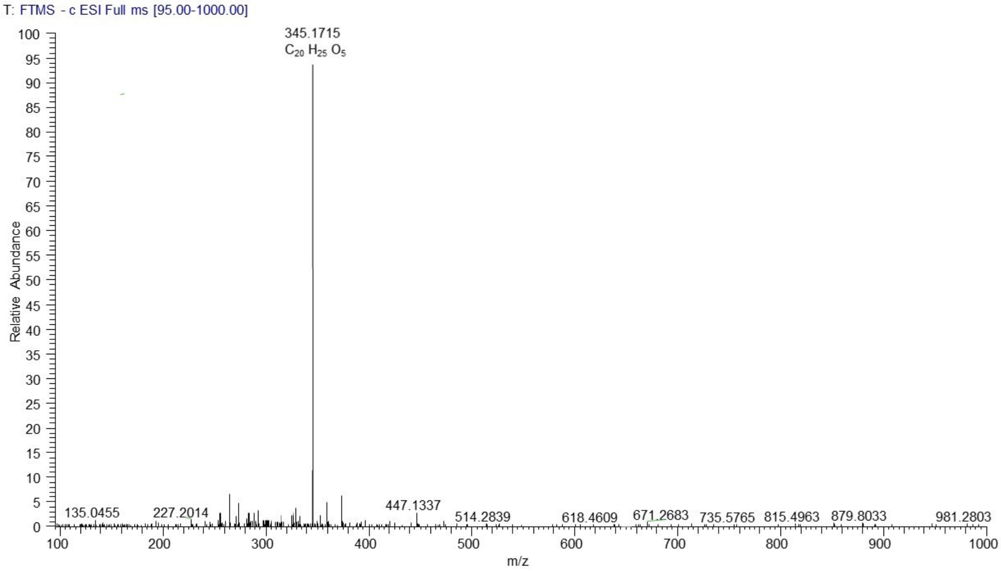

Figure S23. HRMS Spectrum of compound **15**.

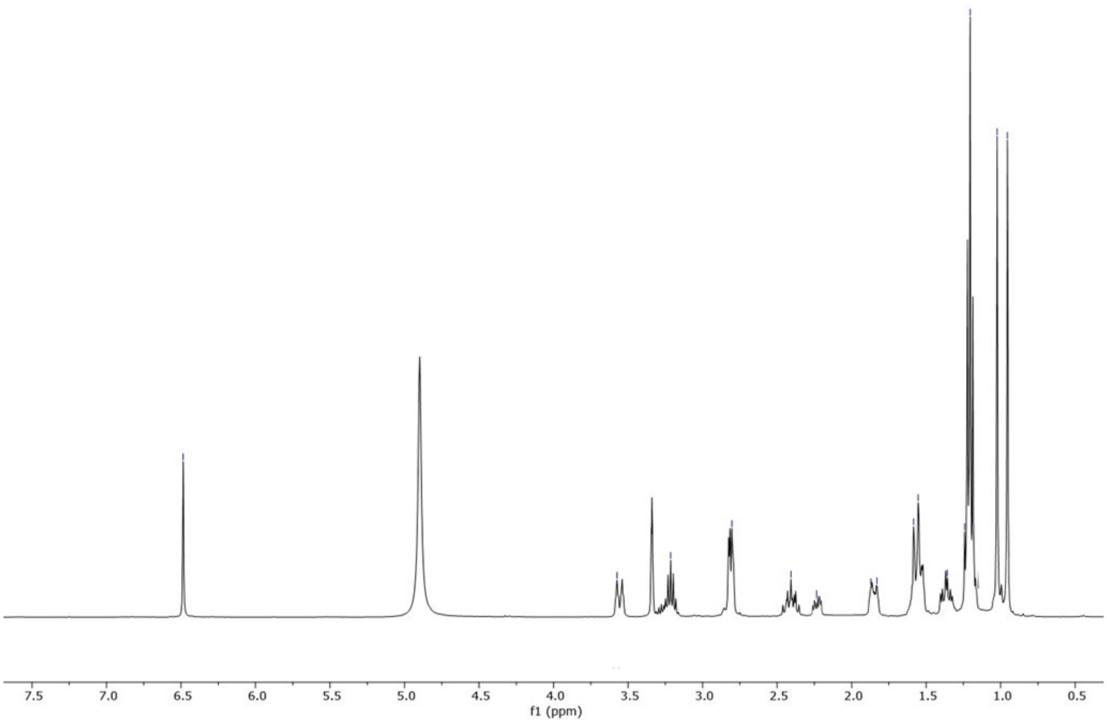

Figure S24. ^{1}H NMR Spectrum (600 MHz, CD_3OD) of compound **16**.

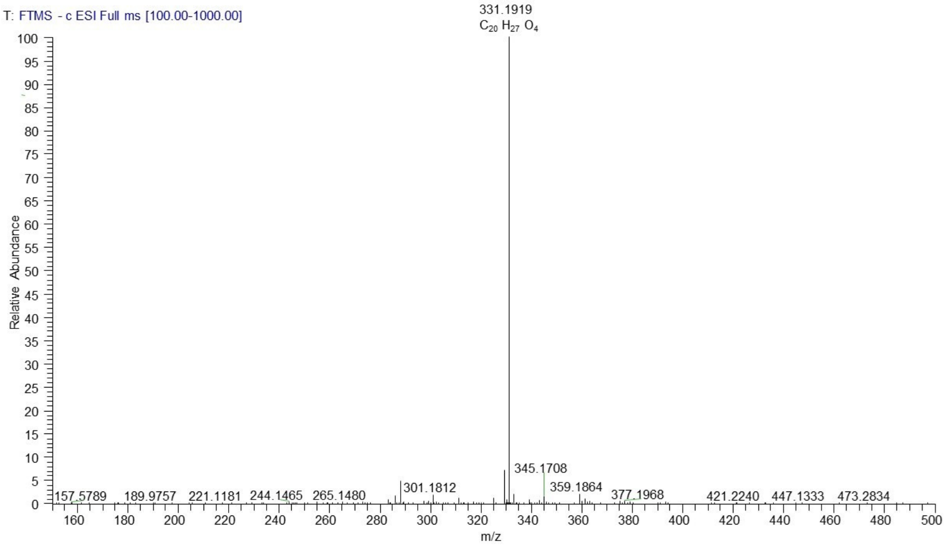

Figure S25. HRMS Spectrum of compound **16**.

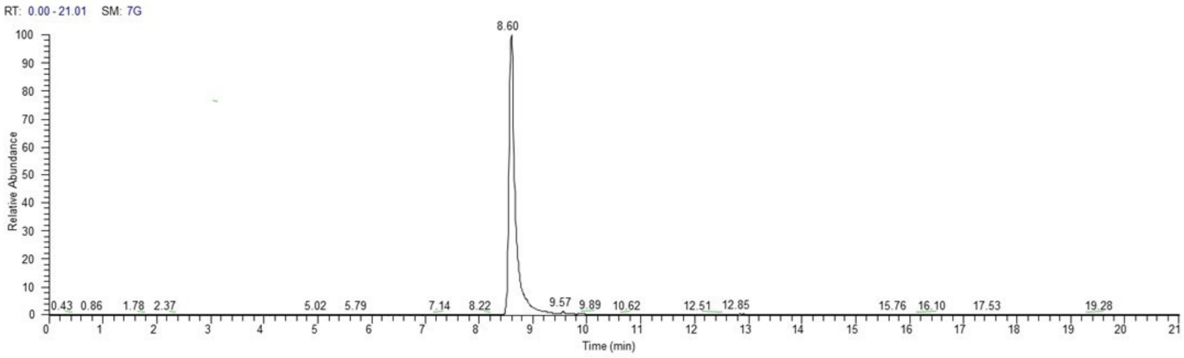

Figure S26. LC-MS Spectrum of compound **16**.

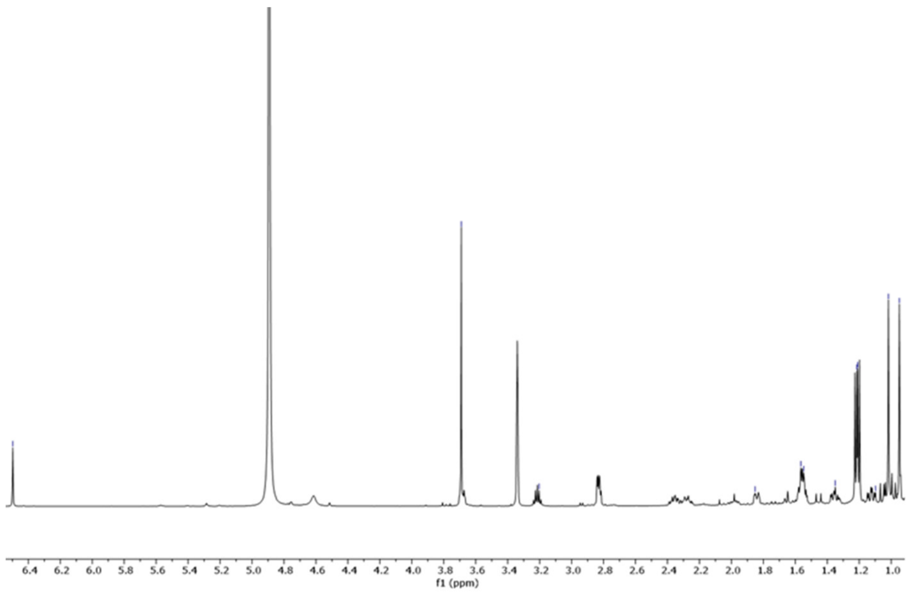

Figure S27. ^{1}H NMR Spectrum (600 MHz, CD_3OD) of compound **17**

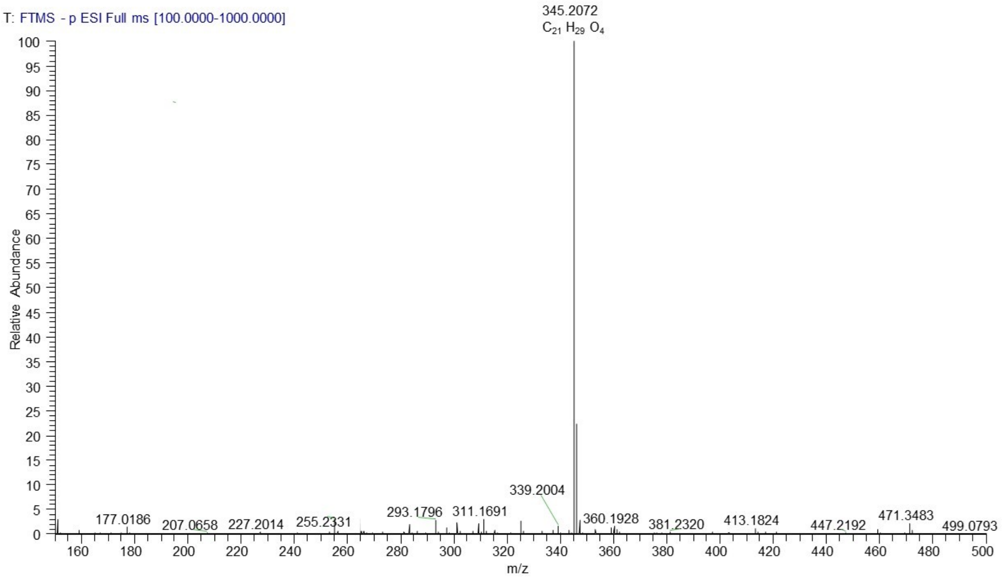

Figure S28. HRMS Spectrum of compound **17**.

Table S1. NMR Spectroscopic Data (600 MHz, CD_3OD) of Compounds **3**, **5**, **7**, **10**, **11**

	**3**		**5**		**7**		**10**		**11**	
position	δ_C, type	δ_H (*J* in Hz)	δ_C, type	δ_H (*J* in Hz)	δ_C, type	δ_H (*J* in Hz)	δ_C, type	δ_H (*J* in Hz)	δ_C, type	δ_H (*J* in Hz)
1	199.0	-	30.0	1.96 m	30.3	2.78 m, 2.57 m	27.9	1.47 m, 2.02 m	28.3	1.96 m, 3.25 m
2	36.5	2.75 m	19.7	1.45 m, 1.58 m	19.9	1.59 m, 1.87 m	19.8	1.52 m, 1.63 m	19.7	1.51 m, 1.26 m
3	37.7	2.06 d (8.5)	41.0	1.21 m, 1.53 m	41.9	1.35 m, 1.51 m	39.4	1.28 m, 1.47 m	34.0	1.26 m, 1.45 m
4	35.1	-	32.7	-	32.8	-	33.3	-	31.3	-
5	153.3	-	55.3	2.28 brs	55.7	1.38 d (4.2)	52.7	2.21 brs	56.1	2.00 brs
6	126.5	7.58 d (8.5)	75.4	4.55 d (3.0)	81.5	4.43 dd (4.3, 4.2)	76.6	4.84 d (3.2)	75.5	5.07 d (2.7)
7	135.1	7.44 d (8.5)	69.22	4.62 d (3.0)	69.2	5.13 d (4.3)	79.4	4.30 d (3.2)	78.4	4.52 d (2.7)
8	127.7	-	128.3	-	128.8	-	130.1	-	127.9	-
9	139.2	-	124.6	-	124.1	-	124.2	-	122.3	-
10	142.3	-	48.2	-	48.7	-	48.2	-	48.0	-
11	173.6	-	142.6	-	143.7	-	143.8	-	143.5	-
12	177.4	-	143.3	-	144.0	-	143.2	-	143.1	-
13	139.7	-	134.9	-	135.6	-	135.9	-	135.7	-
14	123.6	6.48 s	118.3	6.80 s	117.0	6.79 s	121.6	6.79 s	118.7	6.88 s
15	33.3	2.77 sept (6.8)	27.4	3.27 sept (6.8)	27.9	3.28 sept (6.8)	28.6	3.24 sept (6.8)	27.4	3.24 sept (6.8)
16	21.8	1.21 d (6.8)	24.5	1.21 d (6.8)	24.5	1.23 d (6.8)	23.1	1.24 d (6.8)	22.8	1.20 s
17	22.7	1.19 d (6.8)	23.0	1.21 d (6.8)	23.0	1.23 d (6.8)	24.4	1.24 d (6.8)	22.4	1.21 s
18	31.5	1.42	31.5	1.00 s	32.4	1.06 s	32.0	1.06 s	31.5	1.06 s
19	30.2	1.42	22.2	0.92 s	21.3	0.90 s	22.6	0.98 s	22.0	0.88 s
20	-	-	180.2	-	180.5	-	180.1	-	180.3	
7-OCH_3	-	-	-	-	-	-	57.2	3.68 s	60.6	3.63 s

Table S2. NMR Spectroscopic Data (600 MHz, CD_3OD) of Compounds **12, 15-17**

	**12**		**15**		**16**		**17**	
position	δ_C, type	δ_H (*J* in Hz)	δ_C, type	δ_H (*J* in Hz)	δ_C, type	δ_H (*J* in Hz)	δ_C, type	δ_H (*J* in Hz)
1	29.9	2.60 m, 2.86 m	34.0	1.06 m, 3.07 m	35.5	1.23 overlapped, 3.54 ddd (13.8, 3.4, 3.4)	32.2	1.10 m, 3.67 overlapped
2	19.8	1.63 m, 1.97 m	19.2	1.53 m, 2.37 m	19.8	1.83 m, 2.41 m	20.4	1.55 m
3	41.9	1.34 m, 1.52 m	40.6	1.31 m, 1.55 m	42.8	1.37 m, 1.55 m	42.3	1.35 m, 1.55 m
4	34.2	-	34.5	-	34.6	-	34.6	-
5	47.6	1.74 dd (10.6, 5.7)	52.5	1.42 dd (12.0, 1.5)	55.8	1.56 m	55.6	1.44 m
6	29.5	2.21 m, 1.70 m	16.5	1.90 m, 2.06 brs	30.3	1.83 bd (13.3), 2.25 m	30.6	1.85 m, 2.35 m
7	79.3	5.46 d (2.8)	25.4	2.36 m, 2.74 dd (13.0, 7.0)	32.4	2.80 m	32.8	2.83 m
8	131.6	-	146.3	-	130.8	-	131.9	-
9	123.3	-	142.8	-	125.3	-	127.6	-
10	48.5	-	47.5	-	49.5	-	48.7	-
11	143.3	-	178.0	-	143.7	-	154.3	-
12	145.1	-	154.1	-	140.3	-	145.8	-
13	135.6	-	126.3	-	134.5	-	140.2	-
14	112.0	6.72 s	188.0	-	119.2	6.49 s	118.0	6.50 s
15	27.4	3.26 sept (6.8)	27.5	3.20 sept (6.8)	27.6	3.21 sept (6.8)	27.0	3.22 sept (6.8)
16	21.5	1.23 d (6.8)	21.5	1.23 d (6.8)	22.9	1.20 d (6.8)	23.5	1.20 d (6.8)
17	23.1	1.24 d (6.8)	23.1	1.24 d (6.8)	24.2	1.18 d (6.8)	20.2	1.20 d (6.8)
18	31.3	0.91 s	31.3	1.01 s	32.4	1.02 s	33.3	0.95 s
19	19.7	0.91 s	19.7	0.91 s	20.5	0.91 s	23.9	1.03 s
20	180.1	-	176.0	-	180.3	-	180.4	-
12-OCH_3	-	-				-	61.4	3.69 s

Table S3. NMR Spectroscopic Data (600 MHz, CD_3OD) of Compounds **9**, **14**

	9		14	
position	δ_C, type	δ_H (J in Hz)	δ_C, type	δ_H (J in Hz)
1	77.4	3.67 t (2.5)	32.8	1.67 m, 2.35 td (3.8, 14.0)
2	26.3	1.50 m, 2.17 m	18.9	1.50 m, 2.12 m
3	35.7	1.10 m, 1.78 m	41.1	1.62 m, 1.94 m
4	33.3	-	35.8	-
5	52.8	1.70 m	62.2	4.17 s
6	23.4	1.45 m, 1.91 m	202.1	9.65 s
7	36.7	2.61 m, 2.78 m	191.3	9.77 s
8	122.7	-	124.7	-
9	143.1	-	136.7	-
10	74.4	-	49.5	-
11	150.6	-	143.9	-
12	141.2	-	143.2	-
13	135.8	-	133.0	--
14	117.4	6.53 s	131.9	7.59 s
15	27.8	3.24 sept (6.8)	28.5	3.38 overlapped
16	23.0	1.21 d (6.8)	21.6	1.24 d (6.8)
17	23.0	1.21 d (6.8)	22.4	1.24 d (6.8)
18	32.1	0.93 s	32.1	1.50 s
19	22.1	0.96 s	23.6	1.08 s
20	39.7	2.88 d (14.0), 3.10 d (14.0)	178.3	-

Cell Culture

The Caco-2 cell line was obtained from ECACC (Salisbury, UK). Caco-2 cells are derived from human colorectal adenocarcinoma, which, once reaching confluence, spontaneously differentiate into normal enterocytes. Dulbecco's modified Eagle's medium (DMEM) with low glucose and with L-Arginin, phosphate-buffered saline (PBS) without $MgCl_2$ and $CaCl_2$, fetal bovine serum (FBS), and penicillin/streptomycin 1X were obtained from Euroclone (Milano, Italy). Caco-2 cells were grown in T75 flasks until their confluence reached 80%, at 37 °C in a 5% CO_2 humidified atmosphere in DMEM supplemented with 1% antibiotic/antimycotic solution (100 U/mL penicillin, 100 mg/mL streptomycin), and 10% FBS. At passage 45–60, cells were removed from flasks by adding a trypsin solution at 1% and incubating at 37 °C for 5–10 min; Caco-2 cells were then collected, centrifuged, and counted in a Bürker chamber and then seeded into 96-well plates at a concentration of 5 × 10^4 cells/mL for subsequent experiments. Cells were cultured, replacing the medium twice weekly.

MTT Viability Test

To find out any cytotoxic activity of the extract in differentiated Caco-2 cells (21 days post-seeding), viability was estimated using an MTT assay. The cells were seeded in 96-well plates (2.5 × 10^4 cells/mL, 100 µL in each well), incubated with different concentrations (0.5 – 25 µg/mL) of ethanolic extract, with six of the most concentrated compounds (5, 7, 9, 10, 12 and 16, 5 – 250 µM) or with an equivalent amount of ethanol (2.5%) for the controls, and were incubated for 24 h. At 24 h before treatment, the 10% serum supplemented medium was discarded and replaced with a medium with 2.5% serum. After incubation, the medium

was removed, and 100 μL of the MTT solution (5 mg/mL of MTT in PBS in fresh serum free medium, 8% and 92% respectively) was added and left for 6 h at 37 °C. The MTT/medium solution was then removed, and 100 μL of DMSO was added to each well. Afterwards, the absorbance of each well was measured at 570 nm using a microplate reader (Infinite F200, Tecan, Salzburg, Austria). Cell viability was expressed as percentage of control (0 μg/mL) values.

Determination of Intracellular Reactive Oxygen Species (ROS) Production

ROS release in Caco-2 cells was evaluated using the fluorescent probe H_2-DCF-DA.. Cells were seeded in 96-well plates and once differentiated, were incubated with 10 μM of H_2-DCF-DA in 100 μL of PBS for 30 min. Later, H_2-DCF-DA was replaced by the PBS solution containing the ethanolic extract (0.5 - 5 μg/mL) or with compounds 5, 7, 9, 10, 12 and 16 (5 – 25 μM), 30 min prior to adding tert-Butyl hydroperoxide (TBH) 2.5 mM to induce ROS production and lipid peroxidation. Control cells were treated with an equivalent volume of ethanol (2.5 %) in PBS. The increase in cell fluorescence was determined using an Infinite F200 (Tecan, Salzburg, Austria) microplate reader at 485 and 530 nm (excitation and emission wavelengths, respectively). ROS production was monitored by reading the fluorescence emitted every 5 min and after 60 min of incubation.

Statistical Analysis

Data were analysed by means of software GraphPad Prism 5 (GraphPad software, San Diego, CA, USA), using one-way analysis of variance (ANOVA) followed by post-hoc Tukey's test. Levels of $p < 0.05$ were considered statistically significant.

Table S4. LC–HRMS/MS conditions for quantitation of compounds **5**, **7**, **9**, **10**, **12**, **16**

	PRM transition	R2	Regression line	mg/ 100 g dry plant ± SD
**5**	301.18	0.99	$y = 5.85769e^{7}+29857.2x$	20.73 ± 0.14
**7**	301.18	0.99	$y = 3.70495e^{8}+241969x$	11.74 ± 0.39
**9**	179.11	0.99	$Y = 2.03773e^{8}+44704.1x$	91.73 ± 1.87
**10**	283.17	0.99	$y = 1.28006e^{8}+128870x$	1.73 ± 0.40
**12**	285.19	0.99	$y = 5.40847e^{8}+47120.2x$	520.21 ± 23.29
**16**	287.19	0.99	$y = 1.33182e^{7}+1006.25x$	88.16 ± 7.26
